# Risk of second primary malignancies in head and neck cancer patients treated with definitive radiotherapy

**DOI:** 10.1038/s41698-019-0097-y

**Published:** 2019-09-27

**Authors:** Sweet Ping Ng, Courtney Pollard, Mona Kamal, Zeina Ayoub, Adam S. Garden, Houda Bahig, G. Brandon Gunn, Steven J. Frank, Heath D. Skinner, Jack Phan, Joel Berends, William H. Morrison, Jason M. Johnson, Renata Ferrarotto, Erich M. Sturgis, Abdallah S. R. Mohamed, Stephen Y. Lai, Clifton D. Fuller, David I. Rosenthal

**Affiliations:** 1 Department of Radiation Oncology, Division of Radiation Oncology, The University of Texas MD Anderson Cancer Center, Houston, TX USA; 2 Department of Radiation Oncology, Peter MacCallum Cancer Centre, Melbourne, Victoria Australia; 3 The University of Texas Health Science Center, San Antonio, TX USA; 4 Department of Diagnostic Radiology, The University of Texas MD Anderson Cancer Center, Houston, TX USA; 5 Department of Thoracic Head and Neck Medical Oncology, Division of Cancer Medicine, The University of Texas MD Anderson Cancer Center, Houston, TX USA; 6 Department of Head and Neck Surgery, Division of Surgery, The University of Texas MD Anderson Cancer Center, Houston, TX USA

**Keywords:** Head and neck cancer, Cancer epidemiology

## Abstract

Second primary malignancy (SPM) may occur after index head and neck cancer (HNC) treatment. This study evaluated the prevalence and outcome of SPM in patients with HNC treated with definitive radiotherapy. Eligible patients include those with index mucosal HNC treated with definitive radiotherapy between 2000 and 2010. SPM was defined as an invasive cancer at a noncontiguous site diagnosed at least 6 months after completion of radiotherapy. Clinical data were collected, and the Kaplan–Meier method was used to estimate overall survival. In total, 1512 patients were studied. The majority of patients had index oropharyngeal cancer (86%). In all, 130 (9%) patients developed a SPM. The risk of SPM increased exponentially with time with 5-, 10-, and 15-year rates of 4, 10, and 25%. Half of SPMs were within the head and neck or thoracic regions. SPM rates were significantly higher (*p* < 0.0001) in current smokers and former smokers than never smokers with 5-, 10-, and 15-year risk being: never smoker (2, 4, 14%), former smokers with <10-pack year (5, 10, 23%), former smokers with ≥10-pack year (5, 14, 35%), and current smokers (6, 18, 32%). In total, 102 (78%) had subsequent curative-intent therapy. The 5-year overall survival from SPM was 44%. The majority of SPMs were in those with significant smoking history reflecting the same risk factor as for the index mucosal HNC. Nearly one in two patients with SPMs were salvaged underscoring the importance of regular surveillance for SPMs.

## Introduction

Head and neck squamous cell carcinoma (HNSCC) has an incidence of ~50,000 annual cases in the United States, with an annual mortality estimated at 10,000 persons.^1^ With advancements in diagnosis and treatment, the number of head and neck cancer survivors is increasing. The 5-year overall survival for patients with head and neck mucosal cancer treated with radiotherapy is estimated at 65% according to the SEER data from 2008 to 2014.^2^ In the post treatment surveillance setting, we monitor for disease recurrence, late effects of treatment, and second primary cancer. As the risk of index HNSCC disease recurrence decreases exponentially over time, survivorship care focuses on prevention and management of late effects of therapy, and prevention and early detection of second primary malignancy (SPM). SPM may occur after index primary head and neck cancer treatment. Previous studies have reported the incidence of SPMs in a mixed cohort of patients with head and neck cancer treated with or without radiotherapy. The risk of SPM in patients with index head and neck cancer treated with definitive radiotherapy in the contemporary era has not been well documented. Here, we report the prevalence of SPM and subsequent outcomes of patients with SPMs in a large cohort of patients with mucosal squamous cell carcinoma of the head and neck treated with curative-intent radiotherapy.

## Results

### Patient characteristics

From year 2000 to 2010, 1512 patients were eligible for analysis. The median age of the cohort was 55 years (range: 14–87 years). The most common index tumor site was the oropharynx (86%), followed by the larynx (7%). The median radiation dose and fractions delivered were 6996 cGy and 33 fractions. The majority of patients (89%) were treated using intensity-modulated radiation therapy technique. Patients’ and treatment characteristics of the cohort are summarized in Table 1.

**Table 1:** Patients and treatment characteristics

Parameters	*N* (*n* = 1512)	%	SPM (%)
Age	Median = 55 years (range: 14–87 years)		
*Gender*			
Male	1285	84.9	105 (6.9)
Female	227	15	25 (1.7)
*Primary site*			
Oropharynx	1300	86	108 (7.2)
Larynx	105	6.9	13 (0.9)
Nasopharynx	87	5.8	6 (0.4)
Sinonasal	12	0.8	1 (0.1)
Oral cavity	8	0.5	2 (0.1)
*T category*			
T1	396	26.2	28 (1.9)
T2	520	34.4	53 (3.5)
T3	310	20.5	30 (2.0)
T4	231	15.3	16 (1.1)
Tx	55	3.6	3 (0.2)
*N category (all except nasopharynx)*			
N0	172	11.4	17 (1.1)
N1	183	12.1	19 (1.3)
N2a	132	8.7	8 (0.5)
N2b	574	38	51 (3.4)
N2c	219	14.5	17 (1.1)
N3	101	6.7	8 (0.5)
Nx	44	2.9	4 (0.3)
*N category (nasopharynx)*			
N0	16	1.1	0
N1	22	1.5	19 (1.3)
N2	37	2.5	3 (0.2)
N3	12	0.8	0
*AJCC stage*			
I	61	4	7 (0.5)
II	104	6.9	10 (0.7)
III	234	15.5	25 (1.7)
IV	1113	73.6	88 (5.8)
*Smoking status at diagnosis*			
Current	368	24.3	42 (2.8)
Ex-smoker (≥10-pack years)	408	27	48 (3.2)
Ex-smoker (<10-pack years)	199	13.2	17 (1.1)
Never smoker	537	35.5	23 (1.5)
*Radiotherapy technique*			
3D conformal	172	11.4	18 (1.2)
IMRT	1340	88.6	112 (7.4)
Radiation dose	Median = 6996 cGy (range: 5710–7600 cGy)		
Number of radiotherapy fractions	Median = 33 (range: 28–64)		
*Induction chemotherapy*			
Yes	446	29.5	32 (2.1)
No	1066	70.5	98 (6.5)
*Concurrent chemotherapy*			
Yes	1005	66.5	89 (5.9)
No	507	33.5	41 (2.7)

*3D* three-dimensional, *IMRT* intensity-modulated radiation therapy

T and N categories were classified according to the AJCC 7th edition

### Risk of development of a second primary

**Fig. 1:**
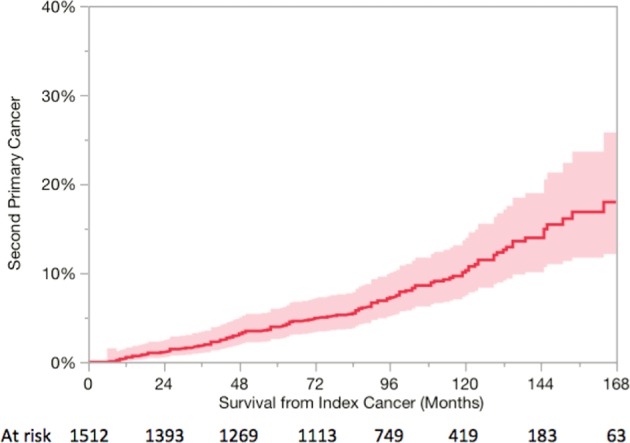
Actuarial risk of developing a second primary malignancy after completion of definitive radiotherapy for index mucosal head and neck squamous cell carcinoma

One hundred and thirty (9%) patients developed a SPM, with 114 patients diagnosed with SPM at least 2 years post treatment. The median time to develop a SPM was 72 months (range: 6–178 months). As depicted in Fig. 1, the actuarial risk of SPM increased exponentially with time with overall rates at 2, 5, 10, and 15 years were 1, 4, 10.3, and 25.2%, respectively.

There was no significant difference in the proportion of patients who developed a SPM according to radiation treatment technique, with 18 (14%) patients previously treated with 3D conformal technique and 112 (86%) had IMRT.

### Smoking status and risk of SPM

**Fig. 2:**
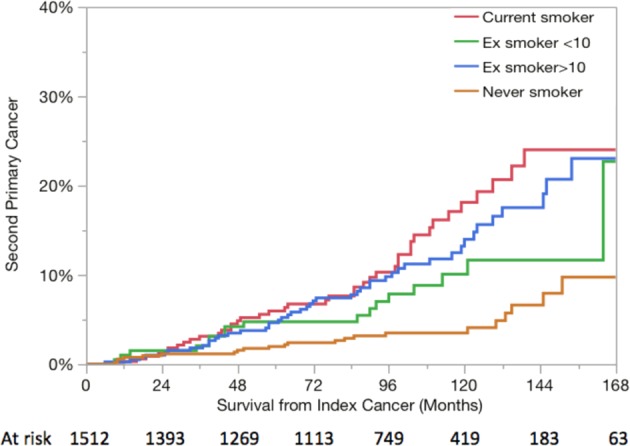
The actuarial risk of SPM according to smoking status at diagnosis of index head and neck cancer

**Fig. 3:**
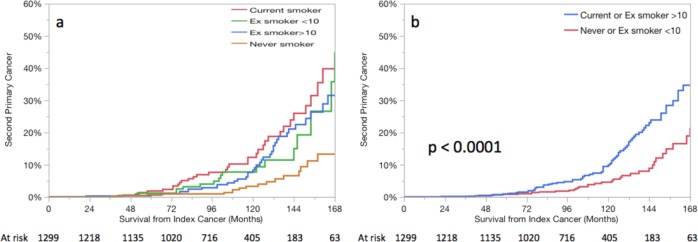
The actuarial risk of SPM according to smoking status at diagnosis for patients with index oropharyngeal cancer. **a** Stratified according to smoking status, **b** stratified into two groups (current or former smoker with >10-pack years versus never or former smoker with <10-pack years)

Of the 130 patients who had a SPM, 90 (70%) had significant smoking history with 42 current smoker and 48 with ≥10-pack year smoking history at the diagnosis of index head and neck carcinoma. Overall, 23 patients were never smoker and 65 were former smoker (17 had <10-pack year history, and 48 had ≥10-pack year smoking history). SPM rates were significantly higher (*p* < 0.0001) in patients who were current smokers and former smokers than never smokers (Fig. 2). The cumulative rates of SPM for each smoking group at 2, 5, 10, and 15 years were: never smokers (1, 2, 4, 14%), former smokers with <10-pack year (2, 5, 10, 23%), former smokers with ≥10-pack year (1, 5, 14, 35%), and current smokers (1, 6, 18, 32%), respectively (Fig. 2). Similar pattern was observed in those with index oropharyngeal cancer with 2-, 5-, 10-, and 15-year cumulative rates of SPM of 0, 1, 5, and 25% for never smokers and former smokers with < 10-pack year, and 2, 11, 10, and 42%) for those who were current smokers and former smokers with > 10-pack year (Fig. 3). On multivariate analysis, patient’s age and smoking status at diagnosis of index cancer and the use of induction chemotherapy emerged as risk factors for the development of SPM (Table 2).

**Table 2:** Multivariate analysis for the risk of development of SPM over time after index cancer

Parameters	Risk ratio	95% confidence interval	*p*-value
Age	17.79	4.6–68.4	< 0.0001
Sex (male)	0.7	0.47–1.14	0.16
Smoking status (current or ex > 10)	2.14	1.47–3.16	< 0.0001
AJCC stage	1.1	0.42–3.14	0.81
Induction chemotherapy (yes)	1.96	1.25–3.02	0.004
Concurrent chemotherapy (yes)	1.46	0.97–2.24	0.08

*P*-values <0.05 are considered significant

### Sites of SPM

**Fig. 4:**
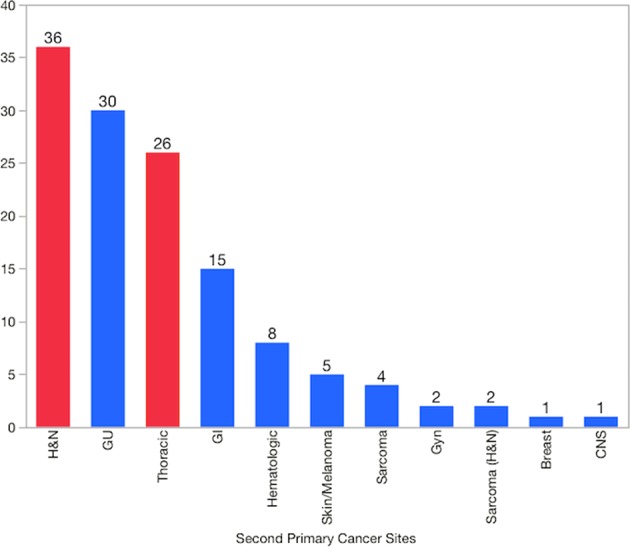
Sites and frequencies of second primary malignancies. Bars in red indicated SPMs within the head and neck, and upper aerodigestive tract (lung/esophagus)

Figure 4 displays the sites and frequencies of SPMs in this cohort. Almost half of the cohort (49%) subsequently developed a SPM within the head and neck, or the upper aerodigestive tract regions—36 (28%) had a second mucosal head and neck carcinoma, 2 (2%) had sarcoma of the head and neck, and 26 (20%) had esophageal (5 patients) or lung carcinoma (21 patients). In those with SPM, 107 (82%) patients were current or former smokers—42 current, 48 former with ≥10-pack year, and 17 former with <10-pack year. Patients who had significant smoking consumption (current and those with ≥10-pack year) comprised 92% of thoracic and 61% of head and neck SPMs.

Genitourinary cancer was the second most common cancer in this group—23 prostate cancers, 3 bladder cancers, 2 renal cancers, 1 urethral cancer, and 1 testicular cancer. Of the five patients with bladder and renal cancers, four patients were current or former with ≥10-pack year smokers. With regards to HPV-related cancers outside the head and neck region, one patient developed cervical cancer 26 months after completing radiotherapy for tonsil cancer. Out of the 130 cases of SPMs, 66 were symptomatic and 64 were detected via routine screening examinations and/or investigations. Table 3 showed the distribution of head and neck SPM in relation to index cancer site.

**Table 3:** Sites of head and neck SPM compared to index head and neck carcinoma site

Index cancer site	Primary site of SPM	Number of cases (*n* = 36)
Tonsil (*n* = 18)	Oral tongue	7
	Larynx	3
	Thyroid	2
	Base of tongue	2
	Parotid	1
	Retromolar trigone	1
	Tonsil (contralateral)	1
Base of tongue (*n* = 14)	Larynx	7
	Oral tongue	3
	Gingiva	2
	Base of tongue	1^a^
	Tonsil	1
Soft palate	Retromolar trigone	1
Nasopharynx	Oral tongue	1
Maxillary sinus	Hard palate	1^b^
Retromolar trigone	Base of tongue	1

^a^Second base of tongue cancer was located away from index tumor location and diagnosed at >10 years from the index cancer

^b^The second tumor was of a different histology than the index cancer

### Clinical outcomes of those with SPMs

**Fig. 5:**
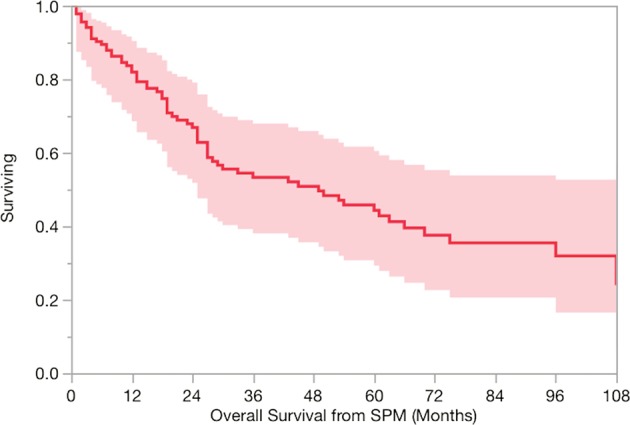
Overall survival from diagnosis of SPM

Overall, the median overall survival for the cohort was 98 (range: 6–199) months. One hundred and two (78%) patients had subsequent curative-intent therapy for SPM. The 2-year and 5-year overall survival from diagnosis of SPM was 67 and 44% (Fig. 5). There was no significant difference in overall survival according to smoking status. The 2- and 5-year overall survival according to smoking status were 85 and 40% for never smokers, 82 and 62% for former smokers with <10-pack year, 64 and 47% for former smokers with ≥10-pack year, and 57 and 35% for current smokers.

## Discussion

In this study, we reported the prevalence of SPMs and the outcomes of those who had SPMs in a large cohort of patients with an index HNSCC treated with curative-intent radiotherapy. Our study highlighted that those with active or ≥10-pack year smoking at the diagnosis of index HNSCC were at significant risk of a subsequent SPM compared with former smokers with <10-pack year or never smokers. The majority of those with current smoking or former smoking status with ≥0-pack year history with SPMs had a second cancer within the upper aerodigestive tract and thorax. Furthermore, the 5-year overall survival in those who developed SPM was 44%, indicating that effective treatment options are available for this group of patients.

This study showed that there was no difference in the proportion of patients who developed SPMs in those who received radiotherapy via 3D-CRT or IMRT technique. Our finding is consistent with previous studies where the technique of radiation delivery did not correlate with the incidence of second primary malignancies.^3^ However, the radiation technique may be associated with the type of second malignancy where 3D-CRT tend to be associated with the development of in-field sarcoma due to the high-dose radiation received by normal tissues. The induction of sarcoma generally require doses of 48 Gy or higher with a prolonged latency period of more than 10 years.^4^ IMRT which delivers dose to the target via multiple treatment fields, on the contrary, is believed to be associated with non-sarcoma second malignancies, as there is a higher volume of normal tissues receiving low-radiation dose compared with 3D-CRT.^3,5,6^ A study investigating second cancers secondary to radiotherapy in the US Surveillance, Epidemiology and End Results (SEER) cancer registries showed no significant relative risk of second cancer for patients who received high-dose radiotherapy for an index head and neck cancer.^7^ In another SEER study, Gao et al.^8^ reported that radiotherapy was significantly associated with 10% increased risk of SPMs in patients with laryngeal cancer, particularly lung cancer. However, this study did not account for smoking status of patients and the location of lung tumors were not described, as radiotherapy fields for laryngeal cancer typically only involved the apices of lungs.

The risk of developing SPMs secondary to radiotherapy remained contentious, as the majority of patients with an index cancer tend to be at higher risk of a second cancer compared with the general population due to their pre-existing risk factors such as lifestyle (smoking, alcohol), environmental, and genetic susceptibilities. As expected, our data clearly reflect the effect of field cancerisation, with almost half of the SPMs occurring within the bronchogenic, esophageal, or head and neck mucosa. In addition, we demonstrated that patients with significant smoking history had higher rates of a second mucosal head and neck, esophageal, or lung cancer. This is of no surprise as the relationship between tobacco usage in patients with index head and neck cancer and subsequent multiple primary cancers has been reported vastly in the literature.^9–15^ Cooper et al.^16^ reported similar findings in 1989 in the Radiation Therapy Oncology Group (RTOG) database, where 110 SPMs were detected in a cohort of 928 patients, with 64% of SPMs arising from the upper aerodigestive tract. Although the rate of SPMs reported in our cohort is similar to historical studies, our cohort had improved 5-year OS of 44% compared with historical studies where 5-year OS was <30%^12,16–18^ potentially reflecting increased physicians’ awareness to screen for SPMs, improved treatment options and efficacy, and success of lifestyle modifications programs, such as tobacco cessation and cancer-screening programs. In addition, cancer chemoprevention is an emerging field, although consensus on the absolute benefit of the drug versus potential side effects of the drug (for example the use of tamoxifen and raloxifene to reduce the absolute risk of breast cancer) remained contentious.^19^ Certain chemoprevention strategies such as the role of aspirin in reducing colorectal cancer risk remained to be proven. However, these options can be discussed and explored with patients at high risk of certain SPM at cancer-screening programs.

More recently, the identification of the human papillomavirus (HPV) as an etiology for oropharyngeal cancer has revolutionized risk stratification for oropharyngeal cancer, with HPV-related oropharyngeal cancer associated with improved survival compared with their non-HPV-associated counterpart.^20^ Although smoking remained a significant risk factor for developing mucosal head and neck cancer, the incidence of the “classic” patient with smoking-related oropharyngeal cancer is on the decline, while the incidence of HPV-related oropharyngeal cancer is on the rise.^21–24^ As those who have HPV-related oropharyngeal cancer tend to be of a younger age demographic, coupled with improved survival, there will be an increase of long-term survivors in several years time. Although HPV/p16 data were not available for our cohort, ~86% had oropharyngeal cancer and 48% of the patients were non-smokers or previous light smokers (<10-pack year history), therefore we may be able to deduce that a significant number of these patients were likely to have an index HPV-related oropharyngeal cancer. Morris et al.^25^ demonstrated that among patients with oropharyngeal cancer, the SPM risk has decreased significantly since 1991 assuming that the majority of these cases were HPV related. As the study is based on SEER database, tobacco use and HPV/p16 status were not available.

In addition to the importance of lifestyle modifications and screening for SPMs within the upper respiratory tract, our study highlights the importance of regular cancer screening in patients with head and neck cancer. Although the primary focus of follow-up after an index head and neck cancer is to monitor the upper aerodigestive tract for recurrence, late effects and SPM, here we showed that a half of our patients develop malignancies beyond the upper aerodigestive tract. Common malignancies such as skin, colorectal, breast, and prostate cancers have effective screening programs. Therefore, we recommend that patients should be referred and enrolled in gender and age-appropriate cancer screening programs. In addition, patients who had a significant tobacco use history should be referred for consideration of lung cancer screening. With early detection and the availability of effective therapies, the cure rates of these SPMs are potentially high.^26^

Our study comes with several caveats. First, there are limitations of a retrospective single institutional study. Second, we reported a strong correlation between smoking status at diagnosis of index HNSCC and subsequent SPM, but we do not have data on the effect of smoking cessation after initial diagnosis on the development of SPM. Third, our study included a heterogenous group of patients and approximately one-third of the group did receive induction chemotherapy. Although our multivariate analysis indicated that induction chemotherapy is a risk factor in the development of SPM, we did not have detailed chemotherapy (induction and concurrent) data and therefore, it was difficult to distinguish the effect of specific chemotherapy agents on subsequent development of SPM. In addition, as the cohort was pre-2010, we do not have p16 or HPV data on those with index oropharyngeal cancer. It is possible that the majority of the oropharyngeal cohort had p16/ HPV-related cancer, as approximately half of the cohort was non-smokers or previous light smokers with <10-pack year smoking history. As the routine reporting of p16 and/or HPV status occurs after the year 2010, and the median time for the emergence of a SPM is ~6 years after completion of treatment for index HNSCC, we are unlikely to have “real-life” data for many more years. Finally, another caveat of the study is that we report on prevalence of SPMs after completion of radiotherapy and some SPM that emerge within the first 2 years after treatment could have been present during index HNSCC diagnosis and treatment at a microscopic level. Nonetheless, to our knowledge, our study is the largest single institutional cohort study that provided the data on the prevalence of SPMs and subsequent outcomes in those who had definitive radiotherapy for index HNSCC.

In this primarily IMRT population, the majority of SPMs were in those with an active smoking status or former smokers with ≥10-pack year history. This study highlights the importance of regular clinical surveillance for SPMs in this population, as almost half of those with SPM can be successfully treated. In addition, tobacco cessation advocacy efforts should continue, and age and/or gender-appropriate cancer screening should not be overlooked in this group of patients during follow-up. In the “HPV epidemic” era, focus on identifying SPM and cancer screening during follow-up is particularly important for those with HPV-related oropharyngeal cancer, as this subgroup of patients have good prognosis and long-term survival.

## Methods

Eligible cases included those treated with definitive radiotherapy for an index head and neck mucosal squamous cell carcinoma at The University of Texas MD Anderson Cancer Center, USA between year 2000 and 2010. SPM was defined as an invasive solid cancer at a noncontiguous site or a hematologic malignancy diagnosed at least 6 months after completion of initial definitive radiotherapy course. This is similar to the Warren and Gates^27^ criteria for multiple primary malignancies:

1. The tumor must be clearly malignant on histology.
2. The tumor must be geographically and/or histopathologically distinct from the index tumor.
3. The possibility of second tumor being a metastasis from the index tumor must be excluded.

Clinical, treatment, and disease data including age at diagnosis, index tumor site and stage, use of induction chemotherapy, radiation dose and fractionation, site of second primary, and subsequent treatment intent of SPM were recorded. Smoking status and tobacco history at presentation of initial cancer diagnosis were also collected. Human papillomavirus (HPV) and p16 status were not routinely reported prior to 2010, and therefore were not collected. This retrospective study was approved by the Institutional Review Board (IRB).

### Statistical analysis

The actuarial risk of developing a SPM was calculated from date of completion of radiotherapy for the index cancer to the date of second primary diagnosis. Overall survival from SPM was determined from the date of second primary diagnosis to date of death from any cause, or date last known alive. Survival outcomes were estimated using the Kaplan–Meier method and groups (smoking status) were compared using the log-rank test. Univariate and multivariate analyses were performed. *P*-value of 0.05 or less was considered statistically significant. All statistical analyses were performed using the JMP package (v 12.1.0, SAS Institute Inc.).

### Reporting summary

Further information on research design is available in the Nature Research Reporting Summary linked to this article.

## Supplementary information

Reporting summary

## Data Availability

The data set generated during and/or analyzed during the current study is available from the corresponding author on reasonable request.
